# Stemming the Rising Tide of Human-Biting Ticks and Tickborne Diseases, United States

**DOI:** 10.3201/eid2604.191629

**Published:** 2020-04

**Authors:** Lars Eisen

**Affiliations:** Centers for Disease Control and Prevention, Fort Collins, Colorado, USA

**Keywords:** ticks, human-biting ticks, tickborne diseases, tick control, disease control, vector-borne infections, vector-borne diseases, control programs, bacteria, parasites, zoonoses, United States

## Abstract

Ticks and tickborne diseases are increasingly problematic. There have been positive developments that should result in improved strategies and better tools to suppress ticks, reduce human tick bites, and roll back tickborne diseases. However, we equally need to address the question of who is responsible for implementing the solutions. The current model of individual responsibility for tick control evolved from a scenario in the 1990s focusing strongly on exposure to blacklegged ticks and Lyme disease spirochetes in peridomestic settings of the northeastern United States. Today, the threat posed by human-biting ticks is more widespread across the eastern United States, increasingly complex (multiple tick species and >10 notable tickborne pathogens), and, across tick species, more spatially diffuse (including backyards, neighborhood green spaces, and public recreation areas). To mitigate tick-associated negative societal effects, we must consider shifting the responsibility for tick control to include both individual persons and professionally staffed tick-management programs.

Ticks and tickborne diseases are distinctly on the increase in the United States (*1**,**2*). Congress responded to this growing problem by establishing a Tick-Borne Disease Working Group in 2016, as part of the 21st Century Cures Act (https://www.fda.gov/regulatory-information/selected-amendments-fdc-act/21st-century-cures-act), and the first biannual Tick-Borne Disease Working Group report was published in 2018 (*3*). Congress also recently passed the Kay Hagan Tick Act (https://www.congress.gov/bill/116th-congress/senate-bill/1657/text/is) to combat vectorborne diseases. Federal public health agencies have generated new strategic plans aiming to strengthen both research and operational capacity to more effectively counter the threat of ticks and tickborne diseases (*4**–**8*). The Entomological Society of America produced a position paper on tickborne diseases (*9*) and led the formation of a new coalition named the Vector-Borne Disease Network, which includes the Entomological Society of America and a wide range of scientific and medical societies, professional associations, and the 5 Centers for Disease Control and Prevention–funded Regional Centers for Excellence in Vector-Borne Diseases (*10*). These are all positive developments expected to contribute improved strategies and better tools to suppress ticks, reduce human tick bites, and roll back tickborne diseases. However, at the root of the growing problem with ticks and tickborne diseases lies the thorny problem of who will be responsible for implementing the solutions.

In the United States, national surveillance of reportable tickborne diseases is achieved through the National Notifiable Diseases Surveillance System (*11*). National surveillance of ticks and pathogens found in ticks was launched only recently as part of the Epidemiology and Laboratory Capacity for Prevention and Control of Emerging Infectious Diseases program of the Centers for Disease Control and Prevention, which provides funding to states, cities, and territories (*12*). The initial focus was on the blacklegged tick (*Ixodes scapularis*) (*13*), with planned expansion to include a wider range of human-biting tick species. Collectively, these national surveillance programs provide information on when and where humans are at greatest risk for exposure to ticks and tickborne pathogens at state and county scales. When risk has been defined in space and time, the next obvious question is how to most effectively suppress ticks, reduce human tick bites, and roll back tickborne diseases. *I. scapularis* ticks and Lyme disease in the northeastern region is perhaps the best example of just how intractable this problem is. In parts of this region, peak risk for exposure to nymphal ticks (the primary vectors of Lyme disease spirochetes to humans) is already clearly defined in space (e.g., shady and moist habitats in backyards, neighborhood green spaces, and recreation areas) and time (spring and early summer) (*14**,**15*). There is no question that every year will be a bad year for Lyme disease in the northeastern region. However, *I. scapularis* ticks and their associated pathogens persist in the environment and continue to cause human illness year after year (*2**,**16*). Potential solutions that have emerged over the past 2 decades include a wide array of approaches to prevent tick bites through personal protection measures or to suppress host-seeking ticks or disrupt enzootic pathogen transmission through environmentally based control methods, but evidence for their impact on human tick bites or illness is limited (*17**–**22*). Moreover, uptake of these solutions by the public remains weak because of limited acceptability of some methods with perceived risk to the environment, pets, or family members, as well as low willingness to pay, combined with the consideration that the lowest-cost methods (e.g., use of tick repellents and daily tick checks) require high levels of daily vigilance over several months each year (*18**,**23**–**25*).

The overall public health threat posed by ticks and tickborne diseases in the United States is steadily increasing to include new human populations because major vector ticks are expanding their geographic ranges (*14*,*26**–**29*), and we are still discovering new native tickborne human pathogens (*1*,*2*,*16*). For public health messaging, surveillance of ticks and their associated pathogens is especially useful at the leading edges of an expanding vector tick species range. Moreover, the negative effect of ticks on human health is expanding from long-recognized pathogen transmission and tick paralysis to also include an allergic response to red meat believed to be associated with previous bites by ticks, including the lone star tick (*Amblyomma americanum*) (*30*). Our most recent warning signal was the introduction and establishment along the Eastern Seaboard of an invasive tick species (the Asian longhorned tick, *Haemaphysalis longicornis*) with potential to negatively impact the cattle industry and perhaps also public health if this tick is found to commonly bite humans in the United States (*31*).

The negative societal effects of ticks and tickborne diseases in the United States, including a general feeling that family members are not safe during outdoor activities in the backyard and elsewhere, has reached the point where we need to rethink the basic concepts of how to counter this threat. We still need a human Lyme disease vaccine (*32*,*33*), and intriguing new tick and pathogen control and tick-bite prevention technologies are on the horizon (*3*,*19*,*20*,*34*,*35*). However, these technologies will still not address the major issue of who should bear the responsibility for implementing proven tick control and tickborne disease prevention solutions. As noted a decade ago by Piesman and Eisen (*36*): “Mosquito control is a community responsibility; tick control is an individual homeowner responsibility. This may explain why currently in the United States, several thousand people are dedicated to mosquito control, whereas only a few dozen are dedicated to public-health related tick control.” Other investigators have more recently similarly noted the difference in how mosquitoborne and tickborne diseases are addressed in the United States and argued for a shift toward area-wide suppression of *I. scapularis* ticks and Lyme disease spirochetes (*37*,*38*). With these considerations in mind, the relentless increase in ticks and tickborne diseases in the United States raises 2 pointed questions that are addressed in more detail in the following sections: First, is it possible to turn the tide of tickborne diseases while control of ticks and their associated disease agents remain an individual responsibility or will this ultimately require a shift to also include a strong community-based effort? Second, can we develop local, professionally staffed programs capable of working with the public to reduce the risk for tick bites on both public and private land?

## Shifting Sands of Ticks and Tickborne Diseases

The concept of tick control as an individual homeowner responsibility emerged, in part, from the knowledge gained about *I. scapularis* ticks, the Lyme disease spirochete (*Borrelia burgdorferi* sensu stricto), and tick encounter locations in the late 1980s and the first half of the 1990s, which made perfect sense at that time. Lyme disease was the near absolute focus among tickborne diseases, most of human infections occurred in the northeastern United States, and residential properties were pinpointed as the most common location for encounters with *I. scapularis* ticks in Lyme disease–endemic areas (*19*,*21*,*39*,*40*). Moreover, as is still the case, both broadcast application of residual acaricides to the vegetation and placement of rodent-targeted tick control devices require physical access for control to be implemented on private properties. The difficulty in accessing these residential high-risk environments presented (and still presents) a major impediment for development of community-driven tick control, and the main focus was therefore on devising tick suppression approaches intended for use in backyards and tick-bite prevention measures for personal protection (*19*). The notable exception was approaches targeting white-tailed deer, which were recognized as dominant hosts for the adult life stage of *I. scapularis* ticks and potentially represent a weak link in the life cycle of the tick (*41*). With the exception of deer fencing, which can be used for single residential properties, deer-targeted tick control approaches (i.e., deer reduction or treatment of deer with topical acaricide) require area-wide implementation to be successful. There is broad consensus that the white-tailed deer is a main driver for the remarkable increase in *I. scapularis* ticks in the northern parts of the eastern United States over the past 40 years (*17*,*19*,*42*,*43*). However, fierce debate continues about the specific thresholds required to be reached for either deer reduction (achieving a sufficiently low deer density) or topical treatment of deer with acaricides (achieving a sufficiently high level of treatment coverage in the deer population) to suppress *I. scapularis* tick populations to the point where we also see an effect on human tick bites and tickborne diseases (*17*,*19*,*43**–**45*). Despite promising results in some studies (*43*,*45*), neither deer reduction nor topical treatment of deer with acaricides has, to date, been widely used operationally to control *I. scapularis* ticks.

In the 25 years since control of human-biting ticks in the United States evolved into an individual homeowner responsibility, the sands of ticks and tickborne diseases have shifted dramatically, and we are no longer facing the same problems as in the 1990s. Although there is empirical evidence that *I. scapularis* tick bites still result most commonly from tick encounters on residential properties in suburban/exurban settings of the northeastern United States (*46*), ongoing spread and population increase of this tick across the northern part of the eastern United States might have resulted in a more spatially diffuse risk for tick encounters as the density of host-seeking *I. scapularis* ticks reached levels across the landscape where even activities of limited duration (compared with the time spent in your own backyard) increasingly results in tick encounters. A recent systematic review and meta-analysis on spatial risk factors for *I. scapularis* tick bites and *I. scapularis* tick–associated diseases in eastern North America concluded that risk occurs in backyards, as well as in neighborhood green spaces and public lands used for recreation (*47*). 

Expanding ranges of other human-biting vector ticks contribute to a changing risk scenario for tick bites. Jordan and Egizi (*48*) reported that during 2006–2016, the vector tick species most commonly collected from humans and submitted to a passive tick surveillance system in New Jersey shifted from *I. scapularis* to *A. americanum*. Both *A. americanum* ticks and the Gulf Coast tick (*A. maculatum*) are spreading northward from their previous core ranges in the southeastern United States (*27**–**29*), and we now also have the invasive *H. longicornis* tick to contend with along the Eastern Seaboard, as far north as New York state (*31*).

Lyme disease is still by far the most commonly reported tickborne disease in the eastern United States, where 2 primary causative agents (*B. burgdorferi* sensu stricto across the eastern half and *B. mayonii* in the upper Midwest) are transmitted by *I. scapularis* ticks (*2*). However, several other tickborne illnesses, as well as co-infections with Lyme disease, are on the rise and increasingly recognized as serious health threats. These illnesses include conditions caused by viral, bacterial, and parasitic pathogens transmitted by *I. scapularis* ticks (*Anaplasma phagocytophilum*, *Babesia microti*, *B. miyamotoi*, *Ehrlichia muris eauclairensis*, and Powassan virus), *A. americanum* ticks (*E. chaffeensis*, *E. ewingii*, Bourbon virus, and Heartland virus), and *A. maculatum* ticks (*Rickettsia parkeri*) (*1*,*16*,*27*,*28*).

In contrast to the situation in the Northeast and upper Midwest, *I. scapularis* ticks are only a minor public health threat compared with *Amblyomma* ticks in the Southeast. Moreover, the potential involvement of *A. americanum* ticks in red meat allergy is concerning because this notorious human-biter is not only abundant in the Southeast but also expanding its range north and thus affecting new human populations (*28*,*29*)*.* Finally, the American dog tick (*Dermacentor variabilis*) remains a threat across its wide geographic range as a vector of the agents causing Rocky Mountain spotted fever (*R. rickettsii*) and tularemia (*Francisella tularensis*) (*1*). Other vector tick species similarly are public health concerns in the Rocky Mountain region and the far western United States, including the western blacklegged tick (*I. pacificus*), the Rocky Mountain wood tick (*D. andersoni*), the Pacific Coast tick (*D. occidentalis*), the brown dog tick (*Rhipicephalus sanguineus*), and *Ornithodoros* spp. soft ticks (*1*).

The strategies devised 2 decades ago to address *I. scapularis* ticks and Lyme disease spirochetes on residential properties in the Northeast are not necessarily well suited to address the current broader, more complex, and spatially diffuse threat of ticks and tickborne diseases in the United States. There is hope that a badly needed human Lyme disease vaccine will be found, but this will only solve 1 part of the overall problem with tickborne pathogens and it will not have any effect on tick populations. Because no silver bullets are on the near horizon to broadly address the increasing threat of ticks and tickborne diseases in the United States, we must reassess the problem and consider new shorter-term solutions.

One reasonable assessment, based on the experience over the past 25 years and the steadily worsening problem, is that the responsibility for tick and pathogen control must be shifted to include both individual persons (responsible for their own properties and use of personal protection measures) and local public health programs with professional staff (responsible for public outreach, assistance to homeowners with selection of appropriate tick control options, and control of ticks and tickborne pathogens in high-use risk areas, such as neighborhood green spaces and picnic areas and hiking trails on public lands). This 2-pronged concept for responsibility should be accompanied by a 2-pronged spatial concept: first, making the backyard a safe, tick-free zone; and second, achieving area-wide suppression of ticks and tickborne pathogens to reduce the risk for tick encounters in other high-use environments.

## Need for Local and Professionally Staffed Integrated Tick-Management Programs

Basic differences in the biology of vector mosquitoes and vector ticks drive the selection of methods and implementation schemes to control these pests. In the United States, local risk associated with tickborne pathogens tend to be predictable both in space and time (across years and seasonally), whereas the local intensity of transmission of mosquitoborne viruses fluctuates dramatically among years and builds over the warm time of the year when mosquitoes are active. This advantage for tick control is counteracted by the fact that mosquito control can focus initially on known larval development sites and then, as needed based on surveillance data, move to a space spray emergency measure not requiring physical access to residential properties. For ticks, every year brings a seasonally predictable emergency situation, risk habitats are diffuse and include both private and public lands, and current options for area-wide tick suppression are limited and have weak evidence bases for impact on human tick bites and disease (*20*). Even control of ticks, such as *I. scapularis* and *A. americanum*, in backyards is problematic because we have a poor understanding of how effectively host-seeking ticks are suppressed across the full extent of a residential property through broadcast of synthetic acaricides, natural acaricides, or fungal control agents by homeowners or commercial pest control companies. A large-scale study that limited application of synthetic acaricide to include only a barrier zone along the lawn–woods ecotone on residential properties did not find the observed suppression of host-seeking ticks within this treated portion of the residential properties to result in reduced human tick bites for the residents (*49*). To more effectively suppress ticks in the environment and reduce human tick bites and tickborne diseases, we need to invest in studies to optimize the effect of existing technologies, as well as stimulate the development of novel approaches.

Nevertheless, elements of organized mosquito control can be used as building blocks for an integrated tick-management program. Well-functioning mosquito management programs are based on the principles of integrated pest management (striving to protect the human population from mosquito bites and mosquitoborne disease agents while at the same time minimizing the impact of pesticides on the environment) and staffed with professionals experienced in public outreach, mosquito biology, pesticide use, and operational surveillance and control concepts. Expanding the activities of existing mosquito management programs to also include ticks (*50*) provides an economy of scale compared with the alternative of having separate community-supported mosquito- and tick-management programs. Specific benefits from building tick responsibilities into an existing mosquito management program might include shared use of existing office/laboratory space, laboratory equipment, and vehicles; presence of professionals already skilled in morphologic vector identification and knowledgeable about basic principles for vector surveillance and control; presence of licensed and highly experienced pesticide applicator personnel; and presence of personnel with previous experience of public outreach for vector-related issues. Regarding access to experienced personnel, effective control of ticks, in backyards or elsewhere, requires control products targeting host-seeking ticks or ticks on host animals to be implemented by persons with a solid understanding of tick biology (e.g., to ensure that the product is applied to the environment in a manner that maximizes contact with host-seeking ticks), the nature of the acaricide product used (e.g., the frequency of acaricide applications needed to provide sustained control over the tick season), and the limitations of the application equipment (which, for example, can effect penetration into microhabitats in which ticks are found). Another potential benefit from strengthening the linkages between mosquito and tick control is an increased involvement by industry in tick control solutions through the already existing interface between industry and the American Mosquito Control Association. A better defined market for tick control products should stimulate industry to invest in new solutions.

The most productive way of exploring the concept of integrated tick-management programs would be (well-funded) demonstration projects focused on geographic locations with strong existing mosquito management programs and severe problems with a wide range of tick species and tickborne diseases. Such an effort is guaranteed to be challenging because it needs to include development of tick-specific knowledge and acquisition of tick-specific equipment; development, implementation, and evaluation of a locally appropriate, standardized tick/pathogen surveillance scheme to address key knowledge gaps, if they exist, for human-biting ticks of local concern and their associated pathogens; development, implementation, and evaluation of a public outreach program to raise local awareness of spatially and seasonally variable risk for exposure to locally found ticks and tickborne pathogens; and development, implementation, and evaluation of schemes for suppression of locally found human-biting ticks on high-use portions of public lands (e.g., along hiking trails, and in and around camp sites, picnic areas, and playgrounds) and on private properties in conjunction with homeowners and using different tick suppression models (e.g., by tick-management program personnel; through contracts with licensed pest control operators from the tick-management program and with oversight by tick-management program personnel; or through homeowner incentives leading to tick suppression executed either by the homeowner or a licensed pest control operator). The lessons learned from such demonstration projects to establish integrated tick-management programs staffed by public health professionals would greatly improve our ability to produce specific and realistic guidance for best management practices.

Moreover, selection of specific tick and pathogen suppression methods to include for either backyard control or area-wide tick management will be challenging because the evidence base for existing approaches is reasonably strong for acarologic outcomes (density of host-seeking ticks and pathogen-infected, host-seeking ticks) but extremely weak for human-based outcomes (human–tick encounters and human illness) (*19**–**21**,**45**,**49*). Initial evaluations of tick and pathogen suppression schemes in an integrated tick-management program would focus on acarologic outcomes; if these were deemed successful, subsequent evaluations should progress to also include human-based outcomes. One major downstream outcome would be improved guidance for best management practices for tick suppression and reduction of human tick bites based on real-world scenarios, which will need to account for local variation in tick species of public health concern needing to be addressed (e.g., only *I. scapularis* ticks, only *A. americanum* ticks, or both species). Cost assessments would be critical to clarify the resources needed to either build ticks into an existing mosquito management program or build an integrated tick-management program from the ground up in settings lacking existing mosquito management programs. Finally, the need for adequate funding for operational tick management cannot be overstated; tick management cannot be incorporated into an existing mosquito management program as an unfunded activity or mandate, and a stand-alone tick-management program equally will require substantial and sustained funding.
